# Integration and evaluation of implementation strategies to improve guideline-concordant bladder cancer surveillance: a prospective observational study

**DOI:** 10.1186/s43058-025-00721-0

**Published:** 2025-04-07

**Authors:** Lisa Zubkoff, A. Aziz Ould Ismail, Laura Jensen, David A. Haggstrom, Soham Kale, Muta M. Issa, Jeffrey J. Tosoian, Mohummad Minhaj Siddiqui, Kennedi Bloomquist, Elisabeth R. Kimball, Susan Zickmund, Florian R. Schroeck

**Affiliations:** 1 Geriatric Research Education and Clinical Center (GRECC), Department of Veterans Affairs, Birmingham/Atlanta VA, Birmingham VA Healthcare System, 700 19th Street S. Birmingham, Birmingham, AL 35223 USA; 2 Division of Preventive Medicine, Department of Medicine, University of Alabama at Birmingham, Birmingham, AL USA; 3 Neal Cancer Center, Birmingham, AL USA; 4 White River Junction VA Medical Center, White River Junction, Hartford, VT USA; 5 VA HSR Center for Health Information and Communication, Roudebush Veterans Affairs Medical Center, Indianapolis, IN USA; 6 Division of General Internal Medicine & Geriatrics, Indiana University School of Medicine, Indianapolis, IN USA; 7 IU Center for Health Services Research, Regenstrief Institute, Indianapolis, IN USA; 8 Geisel School of Medicine at Dartmouth College, Hanover, NH USA; 9 Atlanta VA Medical Center, Atlanta, GA USA; 10 Emory University School of Medicine, Atlanta, GA USA; 11 Tennessee Valley VA Healthcare System, Nashville, TN USA; 12 Department of Urology, Vanderbilt University Medical Center, Nashville, TN USA; 13 Vanderbilt-Ingram Cancer Center, Nashville, TN USA; 14 Veterans Affairs Maryland Healthcare System, Baltimore, MD USA; 15 Division of Urology, University of Maryland, Baltimore, MD USA; 16 IDEAS Center of Innovation, Salt Lake City VA Healthcare System, Salt Lake City, UT USA; 17 Section of Urology and Dartmouth Cancer Center, Dartmouth Hitchcock Medical Center, Lebanon, NH USA; 18 Geisel School of Medicine at Dartmouth College, Lebanon, NH USA

**Keywords:** Implementation strategies, Bladder cancer, Department of Veterans Affairs, Implementation outcomes

## Abstract

**Background:**

Despite guideline recommendations, our prior work revealed more than half of low-risk bladder cancer patients within the Department of Veterans Affairs (VA) undergo too many surveillance procedures and about a third of high-risk patients do not undergo enough procedures. Thus, we developed and integrated implementation strategies to improve risk-aligned bladder cancer surveillance for the VA.

**Methods:**

Prior work used Implementation Mapping to develop nine implementation strategies: change record systems, educational meetings, champions, tailoring, preparing patients to be active participants, external facilitation, remind clinicians, audit & feedback, and a blueprint. We integrated these strategies as improvement approaches across four VA urology clinics. Primary implementation outcomes were qualitatively measured via coding of semi-structured interviews with clinicians and co-occurrence of codes. Implementation outcomes included: appropriateness, acceptability, and feasibility. Exploratory quantitative outcomes included clinicians’ recommendations for guideline-concordant bladder cancer surveillance intervals and sustainability.

**Results:**

Eleven urologists were interviewed. Co-occurrence analysis of codes across strategies indicated that urologists most commonly reported on the acceptability and appropriateness of changing the record system, preparing patients to be active participants (“surveillance grid”), reminders (i.e., cheat sheet), and educational sessions. We confirmed feasibility of all implementation strategies. Urologists indicated that changing the record system had a high impact, reduced documentation time, and guided resident physicians. Preparing patients to be active participants using the “surveillance grid” was seen as an effective but time-consuming tool. Educational sessions were seen as critical to support implementation. In quantitative analyses, clinicians recommended guideline-concordant surveillance about 65% of the time at baseline for low-risk patients, and this improved to 70% during evaluation. Across all risk levels, the largest improvement was observed at site 2 while site 3 did not improve. All sites sustained use of the changed record system, while sustainability of other strategies was variable.

**Conclusions:**

Based on summative interpretation of results, the most appropriate, acceptable, and feasible strategies include changing record systems via a template and educational meetings focused on guideline-concordant surveillance. Future work should assess the impact of the improvement approaches on clinical care processes, particularly on reducing overuse of surveillance procedures among low-risk patients.

**Trial registration:**

The implementation strategies were not considered a healthcare intervention on human participants by the governing funding agency and IRB. Rather, they were seen as quality improvement interventions. Thus, this study did not meet criteria for a clinical trial and was not registered as such.

**Supplementary Information:**

The online version contains supplementary material available at 10.1186/s43058-025-00721-0.

Contributions to the literature

- Targeted implementation strategies are an appropriate and acceptable approach to improve risk-aligned bladder cancer surveillance practices.

- Most strategies were successfully integrated in four hospital sites to promote the use of risk-aligned bladder cancer surveillance.

- Findings from this work show that changing the electronic health record system, combined with education and external facilitation, are the most helpful strategies and may assist others looking to improve risk-aligned bladder cancer surveillance.

- Similar strategies may be helpful in improving guideline-concordant surveillance for other cancers, such as surveillance after treatment for prostate, lung, and colorectal cancer.

## Introduction

Bladder cancer is one of the most prevalent cancers in the Department of Veterans Affairs (VA) [1]. Most patients present with non-muscle invasive “early stage” cancer. After resection, these patients undergo regular surveillance cystoscopy procedures. According to current guidelines [2], the frequency of these surveillance cystoscopy procedures should be aligned with each patient’s risk for recurrence and progression. Risk is categorized as low, intermediate, or high, and is based on bladder cancer history and pathologic details [2]. Our prior work indicated that, despite guideline recommendations, more than half of low-risk patients undergo too many procedures and about a third of high-risk patients do not undergo enough procedures [3, 4].

Thus, we set out to develop and integrate implementation strategies to improve risk-aligned bladder cancer surveillance within the VA. Strategies were selected from 73 strategies clearly defined within the Expert Recommendations for Implementing Change (ERIC) compilation [5]. For this selection, we used a rigorous Implementation Mapping process. In brief, we developed objectives to implement risk-aligned bladder cancer surveillance based on qualitative data organized by Tailored Implementation for Chronic Disease (TICD) framework determinants [6]. We then used data visualization techniques to select strategies with potentially high impact on risk-aligned surveillance (see details in our prior separate publication) [6]. The selected implementation strategies were then combined into four multi-faceted improvement approaches that were subsequently integrated in four VA sites. They included external facilitation, educational meetings, reminders, and preparing patients to be active participants [6].

Here, we present the process of integrating the Implementation Strategies in four VA urology clinics, with the aim to assess the associated implementation and process outcomes, including acceptability, appropriateness, feasibility, urologist satisfaction, fidelity, sustainability, and adoption of risk-aligned bladder cancer surveillance [7].

## Methods

### Overview

In our prior work, nine strategies were systematically developed to improve risk-aligned bladder cancer surveillance guided by the Tailored Implementation for Chronic Diseases framework [6]. The goal was for four VA urology clinics to integrate these nine strategies as specified by the ERIC compilation. The four clinics were identified based on prior quantitative data indicating room for improvement, defined as sites which performed surveillance not aligned with individual patients’ bladder cancer risk [6, 8]. Risk-aligned bladder cancer surveillance thus was the clinical intervention targeted by the implementation strategies. Work conducted at the four sites occurred in three phases: pre-implementation (4–6 months), integration of the strategies (3–5 months), and evaluation (6 months). Integration commenced when we started the first implementation strategy at each site, which was the first external facilitation meeting. Evaluation commenced when sites indicated they had incorporated all the strategies they feasibly could integrate during the 3-to-5-month integration time frame. Sites were actively supported via external facilitation during both the integration and evaluation periods. Sustainment of strategies was assessed 6 months after the end of the evaluation period.

External facilitation was used to support integration, adaptation, and sustainment of all strategies at all sites. Facilitation consisted of at least monthly meetings between the central research team and the local site investigator and their team. Facilitation activities were based on a Blueprint (see Additional file 1 for Blueprint). During each meeting, sites provided an update: successes and challenges were discussed, and then we worked together to address strategy-specific challenges. The expectation for sites was to participate in at least 5 of 16 pre-specified facilitation activities defined by the VA Quality Enhancement Research Initiative [9]. The Consolidated Criteria for Reporting Qualitative Research (COREQ) checklist [10] and the Standards for Reporting Implementation Studies (STaRI) [11] were used for reporting (see Additional files 2 and 3).

### Implementation strategies and implementation processes

We integrated the following nine implementation strategies (labelled according to the ERIC compilation): facilitation, audit and feedback, tailor strategies, conduct educational meetings, identify and prepare champions, remind clinicians, change record systems, prepare patients to be active participants, and an implementation blueprint. Strategies were combined into four multifaceted improvement approaches: external facilitation (including facilitation, audit and provide feedback, and tailor strategies), educational meetings (including conduct of educational meetings, and identification and preparation of a champion), reminders (including changing record systems and remind clinicians with cheat sheets or posters) and prepare patients to be active participants (the only patient-facing improvement approach). Note that in the remainder of this manuscript the term “reminders” refers to the multifaceted improvement approach while “remind clinicians” is a component of that approach, i.e., cheat sheets or posters. Preparing patients to be active participants was operationalized as providing a bladder cancer risk-specific hand-out to patients with a “surveillance grid”, that outlined where they are in the bladder cancer surveillance journey (see Additional file 1 for examples of the surveillance grid). The implementation strategies and improvement approaches were specified as recommended by Proctor including actors, actions, targets of actions, temporality, dose, implementation outcomes likely to be affected, and theoretical justification [6, 12]. They were documented in the implementation blueprint, which was distributed to all participating sites to communicate the components of the improvement approaches. The blueprint also included sections to be filled out by the local site investigator or research coordinator to track progress on strategy integration within each site [6]. For details on strategies see Blueprint in Additional file 1.

Initially, we had planned to implement these strategies at each site over the course of one month. However, while working with the first site we realized that more time was needed, and implementation of the strategies was thus expanded over a 3-month period. All sites initially received facilitation based on the blueprint, which outlined facilitation activities as well as the other seven strategies. The blueprint was shared with the local team at each site at the beginning of the implementation phase and sites were encouraged to implement all strategies. We then supported sites during the integration of the strategies via regular facilitation meetings. During these meetings, the local team was encouraged to follow the steps outlined in the blueprint [6], and the central research team provided advice and support. Sites were free to determine the sequence of integration of the additional seven strategies. Later, topics during the external facilitation meetings transitioned to providing ongoing support and facilitating adaptation.

To characterize the process of integrating the implementation strategies, we categorized strategies into whether they were integrated with a similar timeline at all sites, with variation in the timeline across sites, or not at all sites. A strategy was categorized as successfully integrated when sites reported that they completed the minimum criteria outlined in the blueprint. We cross-checked site-reported data against the central study team’s activity tracking. The timeline was constructed using the notes documented during each facilitation meeting: we determined when each strategy was first discussed and first used at each site. For each strategy, we then plotted the timeline by site. We normalized each timeline to the date of first use to display start-up time needed to launch the strategy.

### Implementation outcome measurement—overview

The goal was to measure implementation outcomes as specified by Proctor et al. [7]. At the design stage, the investigators assessed the most feasible way to measure each outcome. Given the anticipated low number of evaluable clinicians, many outcomes were measured qualitatively. However, quantitative data could be obtained for some outcomes, such as patient satisfaction, fidelity,, and the process outcomes.

### Primary qualitative implementation outcomes

#### Outcomes

The following Implementation Outcomes were measured qualitatively: appropriateness, acceptability, feasibility, and urologist satisfaction. We also collected qualitative data on urologists’ suggestions for improvement and time spent on integrating the strategies. See Appendix Table (in Additional file 1) for further details on outcome measures, definitions, and data sources [7].

#### Participants

We aimed to recruit three urologists from each site, for a total of twelve participants. All those approached agreed to participate, although one never scheduled the interview.

#### Interview procedures

A semi-structured interview guide, informed by Proctor [7], Powell [5], and Weiner [13], was developed by three co-authors (FRS, AAO, LZ) and refined collaboratively with additional co-authors (KB, EK, SZ; see Additional file 1 for interview guide). Three authors (LZ, AAO, FRS) created a priori codes based on key outcomes of interest, including acceptability, appropriateness, usability, feasibility, time spent, and suggestions for improvement. The goal of the interviews was to ascertain which strategies were most and least impactful. We aimed to interview the majority of clinicians who were closely involved in the project and chose to refrain from collecting demographic information as doing so risked participant re-identification.

Interviewees were recruited by local site study coordinators using purposive sampling. A team of qualitative researchers from the Salt Lake City VA Medical Center, who are external from the core study team, conducted interviews and initial coding. Prior to conducting interviews, interviewers (KB: Female, MS, Salt Lake City VA Medical Center, Research Analyst, not related to the central research team; EK: MS, University of Utah, Research Analyst, not related to the central research team) were provided with study details, the interview guide, the preliminary codebook, and samples of implementation materials (e.g., Reminders, Blueprint, Electronic Health Record (EHR) template). Interview participants were provided an information sheet and provided verbal consent to be interviewed and recorded prior to the interview. In addition, the central research team kept notes after each facilitation meeting, documenting the activities the meeting focused on as well as any information provided from each site’s team during these meetings.

The median time of interviews was 21 min (range 16 min – 52 min). Repeat interviews were not conducted because they would not have yielded additional information about implementation outcomes related to the strategies that were already integrated at the time of the interview. Situational factors were not collected. All interviews were one-on-one, audio only, and recorded. Recordings were transcribed verbatim.

#### Qualitative analyses

Anonymized transcripts were transferred to Atlas.ti v23. Transcripts were iteratively analyzed [14]. We deductively coded segments along the two study dimensions: implementation strategy and outcome (see Additional file 1 for codebook). In the first cycle of coding, we used a priori codes (KB, EK) to categorize segments relating to implementation strategy and outcomes [15, 16]. In the second cycle of coding, we sorted segments by code to identify patterns. Commonalities, similarities, or recurring patterns were grouped and summarized (LJ) as themes [17], for each strategy [16–19]. Coding was reviewed collectively, discussed, and agreed upon (LZ, LJ, FRS) throughout analyses. Following the finalization of the coding process, co-occurrence analysis was conducted to provide a frequency count that reflects the number of times participants discussed each implementation strategy and outcome.

### Quantitative implementation outcomes

#### Outcomes

Quantitatively measured outcomes included adoption of risk-aligned surveillance, fidelity, patient experience and patient acceptability, and sustainability.

#### Participants

Participants included site investigators, champions, and patients undergoing bladder cancer surveillance at each site.

#### Data collection procedures

Quantitative data were collected via (1) ongoing tracking of implementation activities by the central research team in a facilitation tracking sheet, (2) site report to the central research team based on the final submitted blueprint, (3) clinician self-report, and (4) chart abstraction. For the chart abstraction, we used national VA Corporate Data Warehouse data to identify patients who recently had surveillance cystoscopy procedures at each site. Trained research assistants then reviewed the electronic charts to abstract each patient’s bladder cancer history, whether bladder cancer risk was documented in the chart, whether a site-specific template was used for documentation, and whether the surveillance recommendation documented in the note was in line with guideline recommendations (for further details see our prior publication) [20].

Patients who presented for surveillance cystoscopy procedures with a history of non-muscle invasive bladder cancer were asked to fill out a one-page pen and paper anonymous survey focused on their experience and on assessing how acceptable the presentation of bladder cancer-related information was during their surveillance visit. This survey was modified based on a prior published survey (see Additional file 1 for survey) [21].

#### Quantitative analyses

We used descriptive statistics for quantitative analyses. For the patient survey, we assessed the overall patient experience based on a single-item response. We calculated an acceptability score based on the four acceptability questions. We converted the mean of the answers to the four questions to a 1- to 7-point acceptability summary scale. We categorized a scale score of 6 or higher as indication for acceptability from the patient’s perspective.

### Exploratory quantitative process outcomes

We measured adoption of risk-aligned bladder cancer surveillance for each bladder cancer surveillance encounter in two ways: (1) whether the clinician accurately assessed bladder cancer risk and (2) whether the clinician recommended a guideline-concordant surveillance interval. Accurate assessment of bladder cancer risk was defined as an encounter note that documented a bladder cancer risk that was in line with the gold standard based on abstraction of pathologic details and prior bladder cancer history [20]. A guideline-concordant surveillance interval was documentation of a recommended follow-up interval within the encounter note that was in line with risk-specific guideline recommendations [20]. In exploratory analyses, we estimated the proportion of encounters with accurate risk assessment and with a guideline-concordant surveillance interval recommendation by study phase (pre-implementation, integration, evaluation). We calculated these proportions overall and stratified by cancer risk status and site. The study was approved by the VA Central Institutional Review Board (CIRB) (No.19–01).

## Results

We supported the integration of nine strategies as part of four improvement approaches across four urology clinic sites within the VA. Sites were located in the east, southeast, and mid-west of the United States (referred to as Site 1, 2, 3, and 4). Qualitative results are based on the interview data from eleven urology clinicians (three female, eight male). Patient survey data was collected from 221 patients. Quantitative process outcomes data was abstracted from encounters for 168 low-risk, 245 intermediate-risk, and 342 high-risk patients.

### Process of integrating the implementation strategies

**Fig. 1:**
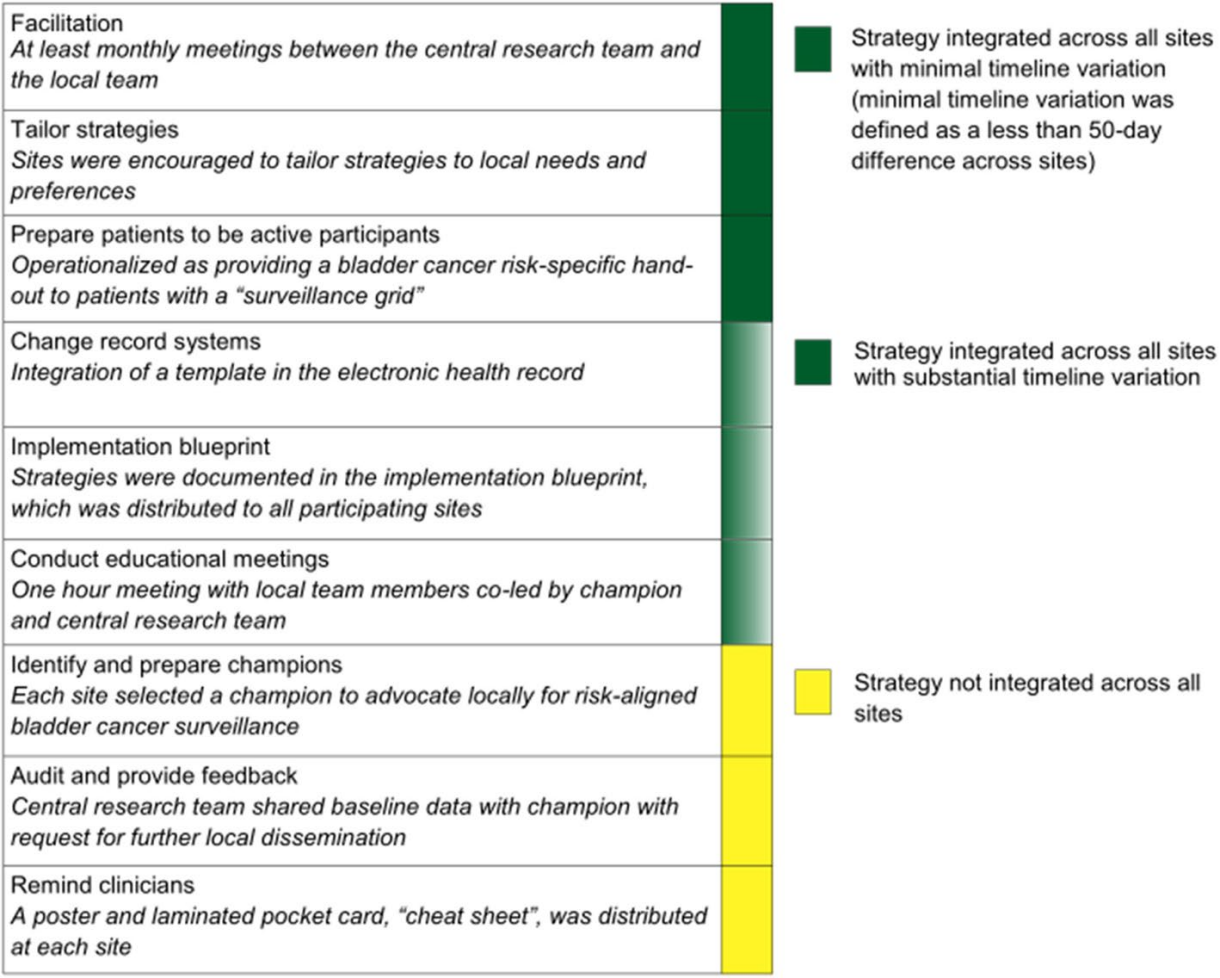
Variation in the timeline of integrating the implementation strategies across four sites. Implementation strategies are labelled according to the Expert Recommendations for Implementing Change [6]

All nine strategies were integrated at least at some sites (Fig. 1). Three of the nine strategies were integrated across all sites within a similar timeline. Another three of the strategies were also integrated across all sites, but with substantial variation in the time needed for integration. When changing record systems across sites, Sites 1 and 3 took more than twice as long (> 6 months) as Sites 2 and 4 (about 3 months). At Site 1, the local team wanted to tailor the draft template to the local context before going live with a change in record systems, and at Site 3 it was challenging to collaborate with the electronic health records team. Time to integrate education meetings also varied substantially, mostly due to the time needed to fit the educational meeting into existing meeting schedules. There was also variation across sites in the time they needed to start tracking integration of the strategies within the implementation blueprint. Appendix Fig. 1 (in Additional file 1) summarizes variation in integration of these strategies.

Three strategies were integrated at only three of the four sites, including champion, audit and feedback, and remind clinicians. Site 1 did not integrate audit and feedback. Site 3 did not integrate a champion and did not integrate all originally planned reminders for clinicians. Challenges with integrating the champion included training, champion engagement with the clinical team at the local site, and a staffing model which limited the champion’s availability to the research team. A challenge with audit and feedback was that one site reported not receiving it, although audit data was provided to them at least once during a facilitation meeting. Lack of wall space was a challenge with integrating reminders via the use of a poster.

### Qualitative implementation outcomes

**Fig. 2:**
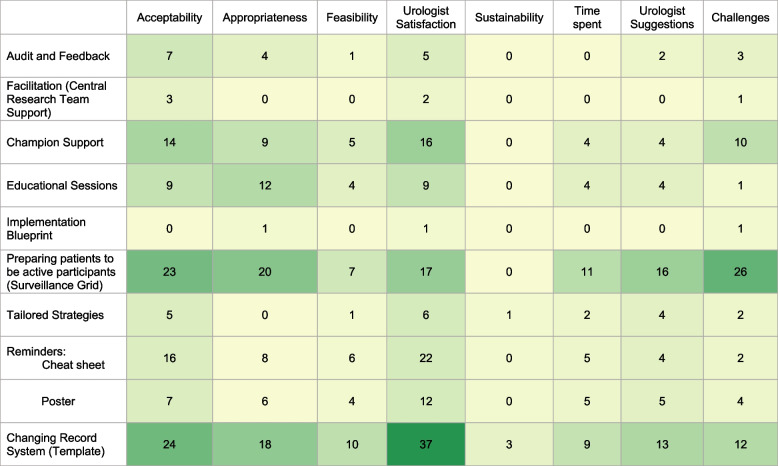
Co-Occurrence Analysis. Each cell represents the frequency count reflecting the number of times participants discussed each implementation strategy and an outcome

Co-occurrence analysis of codes across strategies indicated that interview participants most commonly reported on the acceptability and appropriateness of changing the record system, preparing patients to be active participants (“surveillance grid”), reminders in the form of a cheat sheet, and educational sessions (Fig. 2). Qualitative data indicated feasibility of all implementation strategies, except for facilitation and the implementation blueprint. For those two strategies, we confirmed feasibility using the research team’s tracking of implementation activities. Participants frequently commented on being satisfied with changing the record system, preparing patients to be active participants (“surveillance grid”), reminders, and champion support. Participants frequently reported challenges related to the “surveillance grid” (Fig. 2).

Table 1 presents a summary of qualitative results and themes with exemplary quotes. Participants indicated that changing the record system had a high impact, reduced documentation time, and guided resident physicians. Preparing patients to be active participants by using the “surveillance grid” was seen as an effective tool which supported communication with patients. However, challenges related to the “surveillance grid” included that it was time intensive for clinicians to fill it out and that patients did not bring it back to their clinic visits as originally intended. Reminders in the form of cheat sheets and posters aided in risk stratification and provided visual cues. Educational sessions were seen as critical to support implementation and to overcome implementation barriers. Facilitation was seen as an important component to support implementation.

**Table 1:** Implementation outcomes—summary of qualitative results

Strategy	Themes (in bold) and Exemplary Quotes *(in italics)*
Change the record system (Template)	**Encouraged Risk Stratification and Guideline-concordant Surveillance Recommendations** *"I think familiarizing ourselves with the criteria and the surveillance schedule, you know, reinforcing that has been good. Using the template helps. I think—and it’s particularly good because…the same doctor doesn’t always see the patient. So, it helps to put that in perspective and, you know, where the patient is in the treatment schedule”* [ID01] **Reduced Documentation Time** *"The provider enters the stage, grade, and the size of the tumor, and the focality and the recurrence, and then they can immediately see which risk category this specific patient they have falls into. And then the appropriate surveillance will then pop out, pop up, and then they can follow that"* [ID03] **Guided Residents in Risk-aligned Surveillance** *“I think it’ll like immediately be part of their workload”* [ID09] *“I think that any academic VA center where the residents are doing a lot of independent work in clinic is going to be really reliant on these residents coming through, some of whom may not know oncology super well yet. And maybe they’ve never treated any bladder cancer patients before this. And so previous to having the schedule and the CPRS [EMR] templates they would have to go to a website or to our share drive and look up the guidelines document and like to go page 37 of the guidelines document to find the follow-up protocols for these patients. And that’s a lot of manual work that they’re unlikely to do for every patient. And so being able to easily pull it in through a template I think is an effective strategy”* [ID11] **Required Support from Local IT** *“We are as good as the support we have locally, and that sometimes can be quite challenging with all the demands. We have only one person that does that, and I imagine they are working with the entire surgery and subspecialties, to do all the templates. So that can be a little bit of a challenge, to say the least”* [ID03] **Impact** *“I believe all had an impact. I would say that the most impactful was…CPRS templates”* [ID03] *“I think especially with this, with it being like resident run and we, you know, see these patients who had maybe high-risk bladder cancer with multiple recurrences, and it's managed over the last six years it's just nice having this template that, for the most part, gives you some organization on how to…put all the info together. Gives you, at the top, kind of like the rundown of…what's their risk category, how often should they be followed, when was the last, it asks, you know, the important questions…like when was the last recurrence, last imaging, things like that that can sort of prompt someone to look for the important pieces of information to put their, their story together. So, I think, I think that that's quite nice”* [ID08]
Surveillance Grid (Prepare patients to be active participants)	**Effective Clinician and Patient Tool** *“That was helpful for them to appreciate how often they should come in. I obviously explained it in detail using those cue cards and then at the end I would utilize those sheets that we would hand out to them”* [ID05] *“I think appreciated by patients but also on the provider side…I have personally found it helpful to grab one of those and go over it with the patient. And it just helps to clarify the schedule in your mind. I love the sheets I use them for all the patients because I really like using them and I feel like the patients like seeing what their schedule is and where they are in the process.”* [ID11] *“I think they like it because they can see, especially the high-risk guys they get…three-month cystoscopy and then…when you give them the grid you can show them how long they stay disease free how…the frequency decreased and they’re a little bit encouraged by that. And feel like they, they like it a little bit more to have it in a graphic representation* [ID08] **Time Intensive for Clinicians to Complete** *“Filling out that grid often was pretty time-consuming when I was looking retroactively to see like when they got their previous cystoscopies because I wanted to make sure that they had done all of their appropriate and necessary follow ups. However, after talking with my chairman, he essentially said that, you know, it's okay that I don't fill out all the stuff in their, in their surveillance history on to those grids. Focus more is on what needs to be done next. So just looking like prospectively instead of like retrospectively. The only things that I would write down that were retrospective were the dates of their most recent diagnosis. And if it was readily available, which it is…it fit into the six months, nine months, or 12-month marker, I would, I'd write it down”* [ID05] **Patients did not Return to Office with Surveillance Grid** *“I think the patients just lose them. We haven't seen anyone bring it back”* [ID06]
Remind Clinicians: Cheat Sheet	**Aided in Risk Stratification** *“They made it really clear which patients fall into what category”* [ID11] **Facilitate Surveillance Schedule Concordance between Providers** *“Looking at the sheet and, you know, figuring out where the patient fits into, you know, which category, that’s helpful. So yeah, I think- and it’s particularly good because not-the same doctor doesn’t always see the patient”* [ID01] **Aided Patient Education** *“I utilize the cheat sheets with the patients frequently as I review their particular diagnosis. So I felt that i was very helpful to have a visual aid for them”* [ID05] **Helpful** *“The provider one was very helpful, and I honestly took that around with me. I refer to it frequently”* [ID04]
Remind Clinicians: Poster	**Effective Visual Cue** *“…the provider not only has the grids to give to the patients, but also they can look in every cystoscopy room, we have three of them, and in the hallways, and the cystoscopy office, and in case they want to double-check, they can always just literally turn their head on the wall and they see the poster and they can say: Yes, it is this way"* [ID03] “*The posters and reminders also, similarly, it’s a complicated topic, so having like we have the poster up in our key workroom. And that’s actually really nice because it just provides a reminder of like how one does this, because it has all these facets to it that are hard to remember”* [ID06]
Educational Meetings	**Critical to Implementation** *"I think they were, they were necessary at least to, to review risk stratification for all the different providers. And those are things folks should, should generally know but I, it wouldn’t have made sense to not have those sessions. I think they were helpful" [ID10]* *“The educational sessions help provide awareness to the group of why this is important, and what the ideal output should be” [ID06]* **Overcoming Implementation Barriers** *“We are going to provide you with tools that will make it easier on you. I think they get very happy, and the final, most important part, is to show them the tools and get buy-in"* [ID03] **Suggestions** *“Use more clinical scenarios or try to make them more interactive”* [ID11] *“If there were more education on the front end I think that that is a good idea”* [ID09] **Too Frequent** *“I’m not sure we needed all those meetings but didn’t hurt”* [ID07]
Champion	**Leadership Role** *“Our champion has basically coordinated it so that our bladder cancer surveillance now is grouped individually. So we have clinics specifically devoted to that with providers that are well-versed on the process. So that’s been helpful”* [ID02] **Critical to Addressing Learning Curve** *“I just think there’s a learning curve wit this, and that’s where a repetitive nature, the assistance of the champion, I think until the providers get comfortable if very helpful”* [ID02] **Essential to Implementation and Coordination** *“I think it is important if not critical to have somebody locally as a champion when you launch the project. Someone has to explain it to everyone of like, hey guys we’re doing this and upload the templates and print everything out. And it you didn’t have a local person at all then I don’t think you’d probably be able to run it, to get it started. But once we started it I don’t feel like we ever really had a problem with it where we then had to reach out to our champion again for any problem solving or issues or encouragement. I feel like once we got rolling everyone, it makes sense to people, and for the most part we had pretty good adherence” [ID11]* **Unfamiliar with Role** *“I don’t know who those people are”* [ID09]
Tailored Strategies	**Visual Alignment** *“We aligned the cheat sheet, poster, and grid to have the same streamlined way, the same horizontal-like, timeline, and the same font, the same kind of stuff so that kind of really helped”* [ID03] **Template** “*We modified the templates to include SWOG protocol, BCG schedules and put all the dates in”* [ID09] *“The residents started to modify them a little to add features that they thought were even more helpful that would tell them like when the next cysto was due or when the next BCG was due…I think those things have had a big impact and are continuing to be utilized”* [ID11]
Facilitation	**Important to Implementation** *"Support from the research team was key to just kind of bring this up in the first place and show us importance of like what we're doing and then also like provide some helpful feedback on how to implement this at our site"* [ID06]
Blueprint	**Unrecognized** *“So I assume that's what the blueprint is alluding to. And so I don't recall like this very specific moment that a blueprint was unveiled to us”* [ID06]
Audit & Feedback	**Risk-aligned Surveillance Data not Conveyed by Champion to Broader Team** *“I don’t think I’ve heard any recent data, not from our VA, or from any VA, to tell you the truth”* [ID01] **Group-level Data Would be Helpful to Guide Local Improvement Processes** *"Yeah…I’m a big fan of audit and feedback style approaches. And I think that as long as it’s not, as long as it’s de-personalized and no one feels attacked and it’s across the whole group then I think it can be a good reminder to everyone"* [ID11]

Regarding the blueprints, this was only discussed with the champions, because they were the ones responsible for integration of the strategies. There was a limited number of interviews (*N* = 3) and coding identified confusion among both interviewees and interviewers on the role of the blueprint. Thus, no reliable information could be gleaned from these interviews. However, from the central research team’s tracking of activities, we do know that the blueprint was used as intended to track activities related to the integration of the improvement approaches, suggesting appropriateness and feasibility. Regarding audit and feedback, the central research team distributed information on baseline rates of risk-aligned surveillance at the site to champions, but this information was not reliably shared with local team members at any of the sites. More detailed data on clinicians’ perceptions of the implementation strategies is summarized in Table 1.

### Quantitative implementation outcomes

Table 2 presents quantitative implementation outcomes, including fidelity and sustainability. Across all sites, fidelity ranged from 65% for preparing patients to be active participants to 100% for tailoring. For some strategies, there was variation in fidelity across sites. With regards to sustainability, only the template within the EHR was still in use at all sites 6 months after completion of external facilitation. Sustainability of the other strategies was variable across sites (Table 2).

**Table 2:** Summary of quantitative implementation outcomes assessed during pilot testing. Numbers refer to the numerator and denominator as described in the measure column. For binary outcomes in the sustainability section such as “use of template”, 1 indicates use and 0 indicates non-use. Note: the blueprint is a strategy to present and track the other eight implementation strategies. N/a = not applicable

Strategy	Data Source	Measure	Across all sites	Site 1	Site 2	Site 3	Site 4
**Fidelity**							
Blueprint	n/a	n/a	n/a	n/a	n/a	n/a	n/a
Change record systems (template)	Chart abstraction	# of encounters that have all required components over # of all encounters	218/270 (81%)	13/16 (81%)	36/57 (63%)	116/123 (94%)	53/74 (72%)
Identify and prepare champions	Blueprint items in Sect. 2.4	# of items checked vs # items planned	33/44 (75%)	8/11 (72%)	11/11 (100%)	3/11 (27%)	11/11 (100%)
Tailoring	Blueprint: items 1.4.2a and 1.4.2b	# of items checked vs # items planned	8/8 (100%)	2/2 (100%)	2/2 (100%)	2/2 (100%)	2/2 (100%)
Audit & Feedback	Blueprint items 1.4.5 and 1.4.15	# of items checked vs # items planned	7/8 (88%)	1/2 (50%)	2/2 (100%)	2/2 (100%)	2/2 (100%)
Remind clinicians (Cheat sheet & Poster)	Reported by site in Blueprint items 3.4.1 thru 3.4.3 & 3.4.13	# of items checked vs # items planned	15/16 (94%)	4/4 (100%)	4/4 (100%)	3/4 (75%)	4/4 (100%)
Educational meetings	Blueprint Sect. 2.4	# of items checked vs # items planned	29/32 (91%)	7/8 (88%)	8/8 (100%)	6/8 (75%)	8/8 (100%)
Prepare patients to be active participants (Surveillance grid)	Provider self-report	# of encounters where surveillance grid was used	176/269 (65%)	57/66 (86%)	45/60 (75%)	43/84 (51%)	31/59 (55%)
Facilitation	Blueprint items 1.4.1 thru 1.4.16	Proportion of the 16 activities that were documented as completed	60/64 (94%)	15/16 (94%)	16/16 (100%)	13/16 (81%)	16/16 (100%)
**Sustainability**							
Plan for sustainment^a^	Blueprint	Number of strategies that site plans to sustain	12/15 (80%)	Not collected^b^	5/5 (100%)	2/5 (40%)	5/5 (100%)
Blueprint	Site self-report	Any further use of the blueprint 6 months after external facilitation end	0 (0%)	Not collected^c^	0	Not collected^c^	0
Change record systems (template)	Chart abstraction	Use of template 6 months after external facilitation end (definition: template has required components and is used by at least one provider)	4/4 (100%)	1	1	1	1
Champion	Site self-report	Identified and trained champion exists at site 6 months after external facilitation end	3/4 (75%)	1	1	0	1
Tailoring	Site self-report	Any further tailoring of strategies 6 months after external facilitation end	1/3 (33%)	1	0	Unable to assess	0
Cheat sheet	Site self-report	New providers receive the cheat sheet 6 months after external facilitation end	1/3 (33%)	1	0	Unable to assess	1
Poster	Site self-report	Poster still hanging 6 months after external facilitation end	3/4 (75%)	1	1	0	1
Educational meetings	Site self-report	Use of modules 6 months after external facilitation end (either for new staff or as a refresher)	0/3 (0%)	0	0	Unable to assess	0
Surveillance grid	Site self-report	Surveillance grid is still given to patients by at least one provider 6 months after external facilitation end	1/3 (33%)	1	0	Unable to assess	0

Audit and feedback and facilitation were not offered during the sustainability period

^a^Not collected for champion, facilitation, and tailoring, because at the time of writing of the blueprint, it was felt these were unlikely to be sustained without involvement of the central research team. However, qualitative data indicated that champion and tailoring activities were sustained at some sites as shown in the rows for the individual strategies above

^b^Data was not collected for Site 1; this section was added to the blueprint starting with Site 2

^c^Two sites were not asked about sustained use of the blueprint

In the evaluation phase, we collected 221 surveys from patients presenting for surveillance cystoscopy. Of them, 191 (86%) indicated that the amount of information provided during their bladder cancer surveillance encounter was “just right”. Also, 161 patients (73%) indicated acceptability based on an acceptability scale score of 6 or higher.

### Exploratory quantitative process outcomes

Table 3 presents encounter-level data on accurate documentation of risk assessment and clinicians’ recommended surveillance intervals. For accurate documentation of bladder cancer risk, this improved from 58% during pre-implementation to 75% during evaluation. During pre-implementation, accurate documentation of risk assessment varied by bladder cancer risk group, ranging from 32% for low-risk encounters to 73% for high-risk encounters. When stratified by site, there was substantial improvement in accurate documentation of risk assessment at Site 2, increasing from 27 to 80% (Table 4).

**Table 3:** Encounter-level data on the explorative process outcomes including accurate documentation of risk assessment and clinicians’ recommended surveillance intervals by risk group. N refers to the number of encounters evaluated in each risk group and phase

**Low Risk**			
	Pre-implementation (*N* = 60)	Integration (*N* = 54)	Evaluation (*N* = 54)
Accurate Documentation	19 [31.7%]	25 [46.3%]	45 [83.3%]
Recommendation for guideline-concordant surveillance interval	39 [65.0%]	37 [68.5%]	38 [70.4%]
**Intermediate Risk**			
	Pre-implementation (*N* = 72)	Integration (*N* = 72)	Evaluation (*N* = 101)
Accurate Documentation	41 [56.9%]	40 [55.6%]	63 [62.4%]
Recommendation for guideline-concordant surveillance interval	66 [91.7%]	67 [93.1%]	95 [94.1%]
**High Risk**			
	Pre-implementation (*N* = 116)	Integration (*N* = 87)	Evaluation (*N* = 139)
Accurate Documentation	85 [73.3%]	64 [73.6%]	116 [83.5%]
Recommendation for guideline-concordant surveillance interval	110 [94.8%]	80 [92.0%]	128 [92.1%]
**Overall (combining all risk levels)**			
	Pre-implementation (*N* = 248)	Integration (*N* = 213)	Evaluation (*N* = 294)
Accurate Documentation	145 [58.4%]	129 [60.5%]	224 [76.2%]
Recommendation for guideline-concordant surveillance interval	215 [86.6%]	184 [86.3%]	261 [88.7%]

**Table 4:** Encounter-level data on the explorative process outcomes including documentation of accurate risk assessment and clinicians’ recommended surveillance intervals by site. N refers to the number of encounters evaluated at each site and phase

Overall (combining all risk levels)			
**Site 1**			
	Pre-implementation (*N* = 66)	Integration (*N* = 44)	Evaluation (*N* = 45)
Accurate Documentation	29 [43.9%]	20 [45.4%]	24 [53.3%]
Recommendation for guideline-concordant surveillance interval	54 [81.8%]	36 [81.8%]	39 [86.7%]
**Site 2**			
	Pre-implementation (*N* = 45)	Integration (*N* = 50)	Evaluation (*N* = 55)
Accurate Documentation	12 [26.7%]	25 [50.0%]	44 [80.0%]
Recommendation for guideline-concordant surveillance interval	36 [80.0%]	46 [92.0%]	50 [90.9%]
**Site 3**			
	Pre-implementation (*N* = 84)	Integration (*N* = 58)	Evaluation (*N* = 121)
Accurate Documentation	60 [71.4%]	33 [56.9%]	90 [74.4%]
Recommendation for guideline-concordant surveillance interval	76 [90.5%]	48 [82.7%]	102 [84.3%]
**Site 4**			
	Pre-implementation (*N* = 53)	Integration (*N* = 61)	Evaluation (*N* = 73)
Accurate Documentation	44 [83.0%]	51 [83.6%]	66 [90.4%]
Recommendation for guideline-concordant surveillance interval	49 [92.4%]	54 [88.5%]	70 [95.9%]

Recommendations for guideline-concordant surveillance intervals were already present in more than 85% of baseline encounters and did not change overall (Table 3). When stratified by site, recommendations for guideline-concordant surveillance intervals improved most at site 2, increasing from 80 to 91% of all encounters. At site 3, no improvements in recommendations for guideline-concordant surveillance intervals were observed (Table 4). When stratified by risk level, clinicians recommended guideline-concordant surveillance intervals for low-risk encounters only about 65% of the time during pre-implementation, and this improved to 70% during evaluation (Table 3).

## Discussion

We report on the integration of nine implementation strategies packaged into four improvement approaches for risk-aligned bladder cancer surveillance. Facilitation, tailoring of strategies, and surveillance grids to prepare patients to be active participants were readily integrated at all four sites. Use of an implementation blueprint, the conduct of educational meetings, and changing record systems via templates in the EHR took more than 6 months at some sites. Not all sites were able to integrate a champion, audit and feedback, and all intended reminders. Overall, the implementation strategies were characterized as appropriate, acceptable, and feasible by local clinicians. Clinicians perceived changing record systems with an EHR template and educational meetings focused on guideline-concordant surveillance as impactful. Lack of a fully engaged champion at one site made the integration of strategies and measurement of implementation outcomes challenging. Most participants did not receive audit and feedback data relating to risk-aligned surveillance, indicating that our approach of disseminating baseline data to local teams via the champion was not feasible.

We found that educational meetings and changing record systems are appropriate and acceptable approaches, which is consistent with prior literature. Although clinician education is a common strategy used to change behavior, we recognize it can be difficult to measure its outcomes leading to mixed findings on the effectiveness [22]. Use of a blueprint to present guideline recommendations and implementation approaches was appropriate and feasible, which is consistent with literature showing its applicability to support implementation efforts [23, 24]. Further, a systematic review by Grimshaw et al. found that 73% of the included studies reported use of multi-component strategies and that reminders, educational materials, and audit and feedback were the most evaluated single strategies [25]. Although these strategies changed clinician behavior, the impact on patient outcomes was less clear [26]. Lastly, changing record systems has been shown to facilitate the provision of guideline-concordant care. In a hybrid type I effectiveness-implementation study, Matulewicz et al. used an electronic medical record-based clinical decision support as a strategy to facilitate the use and documentation of evidence-based tobacco screening in VA urology practices, resulting in increased screening at visits [27].

With respect to cancer care, a systematic review by Tomasone et al. found that education, audit and feedback, and reminders for clinicians were the most used strategies. As single interventions, reminders and audit and feedback resulted in improved health care professional behavior (e.g., compliance with the clinical practice guideline, antecedents such as knowledge or attitudes about the guidelines) and patient outcomes (e.g., screening rate, test completion, symptom management, detection of cancer, quality of life) in a cancer care context. When used together as a multi-component strategy as done in this work, group education, reminders, and audit and feedback yielded positive significant outcomes [28].

In our experience, audit and feedback was less successful than frequently reported in the literature [29]. This may be related to several project-specific issues. First, the lack of structured data on both bladder cancer risk and recommended surveillance intervals made data abstraction very labor intensive, which precluded timely delivery and feedback of data. Second, once patients are stratified by cancer risk, month, and site, numbers in each stratum are low which makes it difficult to provide reliable proportions within shorter timeframes. Third, given concerns about confidentiality voiced by participating sites, we refrained from collecting or assessing clinician-level data, which may have made the data that was shared less impactful and relevant for clinicians.

Our data on process outcomes revealed that documentation of accurate risk assessment improved substantially, likely driven by use of the template in the EHR. The largest documentation improvement was seen at site 2, which was likely driven by the lack of a template during pre-implementation resulting in accurate documentation of risk only 27% of the time. However, this did not translate into more guideline-concordant surveillance recommendations, except for a small to moderate improvement among low-risk patients. At first glance, this finding seems to conflict with recent findings reported from our group, where we demonstrated an association between accurate documentation and guideline-concordant surveillance recommendations. However, further informal discussion with the site that had most of the guideline-discordant surveillance recommendations among low-risk patients revealed that there was a misinterpretation of the recommendations that were included in the template for low-risk patients. This issue contributed to a decrease in guideline-concordant surveillance recommendations among low-risk patients after integration of the template. This problem could easily be addressed with targeted education and by adapting the template to decrease the risk for misinterpretation. The template was also highly valued by the participants in our interviews. Thus, integration of a template into the EHR seems to be one of the most promising implementation strategies for guideline-concordant bladder cancer surveillance but should be combined with appropriate educational meetings. This tool may be of particular help to increase surveillance for high-risk and reduce or de-implement unnecessary services for low-risk patients.

### Limitations

The data on integration and implementation outcomes rely on site reporting. However, when we compared the data to tracking done by the central research team, there were no pertinent differences.

Although we conducted interviews with clinicians from all sites, we had a limited sample and therefore cannot state that we reached saturation. Our study was not powered to assess for statistically significant differences in surveillance recommendations or process outcomes, given the anticipated low number of patients per risk category and site. Thus, no definitive conclusions can be drawn on whether the implementation strategies improved guideline-concordant surveillance recommendations among low-risk patients and whether they would contribute to less overuse of cystoscopy procedures in this population. In addition, the baseline rate of approximately 85% guideline-concordant surveillance recommendations across all risk categories combined (Table 2) was higher than suggested by our prior preliminary data [3, 4]. This was likely due to secular trends that happened while the earlier phases of this multi-year project were completed. As such, there was little room for improvement – especially among high-risk patients. Future work should focus on improving guideline-concordant surveillance recommendations for low-risk patients, as about a third of them were issued recommendations for too many surveillance cystoscopy procedures (Table 3). Finally, we may not know the impact of each individual implementation strategy because we asked participants about the most and least impactful strategy, thereby potentially missing important thoughts on strategies that fall in the middle.

### Implications for Practice

Despite these limitations, our findings have implications relevant not only for surveillance after bladder cancer treatment, but also for surveillance after treatment for other cancers. For example, the frequency and type of surveillance for patients who underwent treatment for prostate, lung, or colorectal cancer also depends on factors influencing their risk for recurrence and progression [30–32]. These factors may include stage, grade, and type of treatment received. Thus, clinicians need to assess cancer risk for these patients and provide guideline concordant recommendations for surveillance intervals after treatment. The implementation strategies evaluated in our current work might also be applicable and further tailored to clinicians who manage these and other cancers.

## Conclusions

Based on a summative interpretation of our results, the most appropriate, acceptable, and feasible, strategies include changing record systems via an EHR template and educational meetings focused on guideline-concordant surveillance. Identifying and preparing a champion at each site was critical for integration of the strategies and for the collection of implementation outcomes. It was time consuming to provide surveillance grid handouts to patients. However, surveillance grids and external facilitation may enhance the effectiveness of the other strategies. Tailoring of strategies should be allowed, provided core components of each strategy are maintained. Further research should assess the extent to which a broader integration of these strategies improves guideline concordant surveillance for low-risk early-stage bladder cancer patients and the extent to which adding patient-facing surveillance grids and external facilitation enhances the strategies’ effectiveness.

## Supplementary Information

Additional file 1.

Additional file 2.

Additional file 3.

## Data Availability

The data sets generated and analyzed during the current study are not publicly available because they contain potentially identifying and sensitive information but are available from the Principal Investigator on reasonable request. Upon request from a qualified investigator, a limited data set will be created for that investigator’s use and shared pursuant to a Data Use Agreement (DUA) appropriately limiting use of the data set and prohibiting the recipient from identifying or reidentifying (or taking steps to identify or reidentify) any individual whose data are included in the data set. Investigators who request to use the data will be required to obtain institutional review board approval and sign the DUA before release of the data. Interested investigators are encouraged to directly contact the Principal Investigator, Florian R. Schroeck.

## Abbreviations

CIRB: Central Institutional review board
Cysto: Cystoscopy
EHR: Electronic health record
ERIC: Expert recommendations for implementing change
QUERI: Quality enhancement research initiative
TICD: Tailored implementation for chronic diseases
VA: Department of Veterans Affairs
