# Supplementary material for: Integration and evaluation of implementation strategies to improve guideline-concordant bladder cancer surveillance: a prospective observational study
Source: Implement Sci Commun. 2025 Apr 7;6:37. doi: 10.1186/s43058-025-00721-0 (PMC11977926; doi:10.1186/s43058-025-00721-0)

**Additional File 1**

1. **Blueprint for Improvement Approaches Developed for ‘Implementing Risk-aligned Bladder Cancer Surveillance (ImpRaBS)’**
2. **Examples of the “Surveillance Grids” for low-, intermediate-, and high-risk bladder cancer**
3. **Appendix Table**
4. **Interview guide for clinical staff interviews in Aim 3 ImpRaBS**
5. **Codebook**
6. **Survey collected from Veterans in Aim 3**
7. **Appendix Figure 1**

**1. Blueprint for Improvement Approaches Developed for ‘Implementing Risk-aligned
Bladder Cancer Surveillance (ImpRaBS)’**

**Principal Investigator:** Florian R. Schroeck, MD, MS

**Site:**

**Site PI:**

**Champion:**

**INTRODUCTION:**

Bladder cancer is one of the most common cancers in the VA. Most patients are diagnosed with non-muscle invasive bladder cancer (NMIBC) and then undergo long-term surveillance cystoscopy procedures.

VA data leading up to this project has shown that many Veterans with low-risk NMIBC undergo too many surveillance cystoscopy procedures and those with high-risk NMIBC undergo too few surveillance cystoscopy procedures.

**Goal of this project:**

The overarching goal of this project is to get the appropriate frequency of surveillance cystoscopy procedures to all Veterans, according to their individual bladder cancer risk. To do this, we have developed improvement approaches that we are now piloting at your site.

**How to use this document:**

This document provides detailed information on the improvement approaches. It can be used by local team members to:

- obtain descriptions of the improvement approaches
- document progress on the improvement approaches
- document any modifications that are being made to the improvement approaches

**Methods used:**

Over the last 2 years, we have collected data from your site as well as other VA sites on barriers and facilitators of appropriate frequency of surveillance cystoscopy. These were systematically analyzed. Based on that data set, we then developed a set of nine strategies that have been grouped into four improvement approaches. We are now eager to determine the extent to which these help your site provide the recommended appropriate frequency of surveillance cystoscopy.

The four improvement approaches are:

- External facilitation
- Educational meetings
- Reminders
- Prepare patients to be active participants

**Timeline of pilot testing:**

| Task | Time |
| --- | --- |
| Discussion with the site principal investigator |  |
| Implementation of the improvement approaches |  |
| Providing support to the local team and collect data |  |
| Ongoing improvement and sustainability (see page 18 – last section) |  |

**Our team:**

We are a team of urologists, implementation scientists, and data experts, funded via a VA Health Services Research and Development Merit grant to conduct this project. The principal investigator is Florian R. Schroeck, MD, MS. The Implementation Science lead is Lisa Zubkoff, PhD. The project manager responsible for all data related aspects is A. Aziz Ould Ismail, MD, MS.

**Contact Information for the Central Research Team:**

**Florian R. Schroeck, MD, MS, Principal Investigator:**

[florian.schroeck@va.gov](mailto:florian.schroeck@va.gov);

**Lisa Zubkoff, PhD, Lead Implementation Scientist:**

[Lisa.zubkoff@va.gov](mailto:Lisa.zubkoff@va.gov)

**Aziz Ould Ismail, MD, MS, Project Manager:**

[AbdolAziz.OuldIsmail@va.gov](mailto:AbdolAziz.OuldIsmail@va.gov);

**EXTERNAL FACILITATION:**

**What is happening?** Our team is going to work with you (as local leaders and champions) to implement and adapt the proposed improvement approaches to best fit your local needs. This will entail an iterative process during which we will interact with the site PI or champion, support implementation, share materials and data, and collect and incorporate your suggestions.

**Why is it happening?** For the proposed improvement approaches to be successful at your site, we believe that your local team will benefit from guidance and support from our team. Also, our team will help you modify improvement approaches to match local resources and workflow. We will share data on appropriate frequency of cystos for 6 months after implementation.

**Where is this happening?** Facilitation will occur in virtual meetings or via email as needed.

**When will this happen? And for how long?** One pre-implementation meeting, then (starting in implementation month) every other week meeting for 8 weeks, followed by meeting every four weeks thereafter. The goal is to continue these meetings for at least 6 months after the month of implementation.

**How is this happening?**
In this section, we will specify the tasks that our team will do and the ones that you are expected to carry out at your local site. We will specify the items that you must complete to meet the minimum criteria for implementation of this improvement approach.

1. **What would we like to accomplish?**

Using this improvement approach, we want to accomplish goals at the facility, local team member, and patient levels:

1. Facility level:

- Help you learn about the facility-level rate at which urology attendings / urology residents / Advanced Practice Providers assess and document bladder cancer risk and assign the appropriate frequency of cystoscopy procedures
- Help with tailoring improvement approaches to local needs and preferences
- Help with using available data to support requests for necessary resources (e.g., staffing, space, teleurology, etc.)

1. Local team member level:

- Support local teams in identifying barriers and troubleshoot them, including sharing and obtaining necessary resources
- Support role-appropriate tasks as suitable for each site (e.g., nurses may do some tasks at some sites, but urology attending / urology resident / Advanced Practice Providers may do them at others)

1. Patient level:

- Support sites with preparing patients to be active participants
- Incorporating patient scheduling into the improvement approaches may be appropriate for some sites but not for others

1. **How are we going to help your site meet these goals?**

- Regularly discuss with site PI/champion implementation of the improvement approaches and how to tailor proposed improvement approaches to fit local needs
- Assist the site PI/champion with troubleshooting local implementation challenges
- Support standardized documentation of modifications made to the improvement approaches
- Share data about assessment and documentation of bladder cancer risk and urology attendings / urology residents / Advanced Practice Providers’ assignments of appropriate frequency of cystoscopy procedures
- Deliver the data in user-friendly format, e.g., appropriate plots, to the site PI/champion

1. **Who is the key contact and regularly participating in facilitation at your site?**Note: Delegate can be used for specific tasks, for example if tailoring or data review are assigned to specific team members.

Site PI  Champion
 Delegate [please specify who and for what task]:

1. **As part of facilitation your site may participate in multiple activities.
   Please identify which activities occurred at your site [check all that apply]:**

Participated in pre-implementation planning meeting(s) [1]

Shared modifications to improvement approaches with central research team [2a]

Documented suggested modifications in the blueprint [2b]

Completed administrative tasks (e.g., scheduling meetings) [3]

Provided updates and feedback on progress, successes, and challenges to central research team [4]

Reviewed data to assess baseline facility-level rate of appropriate frequency of cystoscopy procedures [5]

Discussed and clarified roles and responsibilities and what will occur [6]

Identified key local stakeholders and engaged them to obtain their buy-in [7]

Discussed structural change (e.g., staffing, how patients move through the system, etc.) with central research team or local team [8]

Set clear, realistic goals and priorities for improving bladder cancer surveillance [9]

Selected local change agents (e.g., champions, opinion leaders) for improving bladder cancer surveillance [10]

Managed local group dynamics or team processes to improve bladder cancer surveillance [11]

Identified local problems around bladder cancer surveillance [12]

Solved local problems around bladder cancer surveillance [13]

Received ongoing support from the central research team [14]

Received updates and feedback, including data feedback from the central research team [15]

Discussed and planned the extent to which local site will sustain the implemented improvement approaches [16]

1. **Who would benefit from this improvement approach?**

- Champion/site PI
- Urology attendings / urology residents / Advanced Practice Providers, nurses, and schedulers
- System

1. **We will track your participation in facilitation while we deliver it.**We expect that your site will participate in at least 5 of the activities listed under Section 4 above. We recommend that every site at least:

- Participated in pre-implementation planning meeting(s)
- Shared modifications to improvement approaches with central research team
- Documented suggested modifications in the blueprint
- Reviewed data to assess baseline facility-level rate of appropriate frequency of cystoscopy procedures
- Received updates and feedback, including data feedback from the central research team

1. **Please list any tasks not listed above (i.e. modifications) you have taken while implementing this improvement approach** **(What? Why? Where? When? Who? How?):**

Observed benefits or drawbacks of tasks not listed above (i.e. modifications) while implementing this improvement approach:

**IDENTIFYING CHAMPIONS AND CONDUCTING EDUCATIONAL MEETINGS:**

**What is happening?** Our team is going to work with you (as local leaders) to identify a champion. The champion will advocate locally for getting patients with non-muscle invasive bladder cancer the appropriate frequency of surveillance cystoscopy procedures. The champion will lead or co-lead educational meetings to improve the local team members’ knowledge about appropriate frequency of surveillance cystoscopy procedures.

**Why is it happening?** The on-site champion can help deliver/fine-tune our proposed improvement approaches to best fit the local needs. Also, champions serve as a bridge between the clinicians at your site and the central research team. The champion will lead educational meetings at your site to provide information about the guidelines for non-muscle invasive bladder cancer and the improvement approaches.

**Where is this happening?** Your site will designate a champion, who will identify a space for the educational meetings.

**When will this happen? And for how long?** The champion at your site will be identified by:      . Educational meeting(s) at your site are scheduled for:      .

**How is this happening?**
In this section, we will specify the tasks that our team will do and the ones that you are expected to carry out at your local site. We will specify the items that must be completed to meet the minimum criteria for implementation of this improvement approach at your site.

1. **What would we like to accomplish?**

- Raise awareness of guideline recommendations on appropriate frequency of surveillance cystoscopy procedures (e.g., AUA/NCCN)
- Clarify guidelines and promote local discussions about risk assessment and appropriate frequency of surveillance cystoscopy procedures
- Create/promote a culture in which team members (Urology attendings / urology residents / Advanced Practice Providers, Nurses, Schedulers) collectively adhere to the guidelines and collaborate to assign appropriate frequency of surveillance cystoscopy procedures
- Review data and literature that support guideline-concordant care with clinicians
- Champion troubleshoots barriers to provide appropriate frequency of surveillance cystoscopy procedures
- Champion articulates that appropriate frequency of surveillance cystoscopy procedures is high priority area for all team members
- Champion clarifies team members’ roles in assigning appropriate frequency of surveillance cystoscopy procedures

1. **How are we going to help your site meet these goals?**

- Develop educational modules about the guidelines for non-muscle invasive bladder cancer (Module #1) and the improvement approaches (Module #2)
- Post educational modules to be available to local team members
- Provide and train the champion on resources (educational materials, templates, surveillance grids for patients, blueprint) that help raise awareness about appropriate frequency of surveillance cystoscopy procedures
- Communicate with the champion and address their concerns/feedback to minimize their workload

1. **The following individual was identified as the champion at your site:**       **Who’s in charge of implementing the educational meetings at your site?**

Site PI  Champion  Delegate [please specify who and for what task]:

1. **Tasks to complete at your site to meet the goals of this improvement approach:
   Please identify which activities occurred at your site [check all that apply]:
   For educational meetings:**

Designate time, location, and frequency for the educational meetings

Designate time for the clinicians to participate in the educational meetings

Notify the clinicians about the educational meetings and follow up with reminders

Engage clinicians in discussions of guidelines and report feedback to the central research team

Lead or co-lead the educational meeting

Assure that new local team members (e.g., residents) will have access to education

Offer refresher training to local team members

Encourage team members to talk about integrating the improvement approaches into local workflow

**For Champion:**

Is trained by Site PI or central research team on use of the Blueprint and the improvement approaches

Raise awareness (e.g., in educational meetings, Module #1) about the guidelines

Articulate that appropriate frequency of surveillance cystoscopy procedures is high priority area for all team members and that it is consistent with the VA National Oncology Program’s aim to provide "standardized, evidence-based disease management and treatment protocols" (<https://www.cancer.va.gov/CANCER/docs/NOP_Brochure_vFinal_DIGITAL.pdf>). (e.g., in educational meetings, Module #1)

Review improvement approaches (Module #2) with local team members (can be done in educational meetings)

Provide access to the guidelines for appropriate frequency of surveillance cystoscopy procedures

Train urology attendings / urology residents / Advanced Practice Providers on using templates/surveillance grids/educational materials (e.g., in educational meetings)

Help with troubleshooting, and report to the central research team for assistance as needed

Discuss the appropriate frequency of surveillance cystoscopy procedures with urology attendings / urology residents / Advanced Practice Providers (e.g., in educational meetings)

Encourage urology attendings / urology residents / Advanced Practice Providers to change existing frequency of surveillance cystoscopy procedures to be consistent with guideline recommendations (e.g., in educational meetings)

Recruit advocates for appropriate frequency of surveillance cystoscopy procedures

Report feedback to the central research team

1. **Who would benefit from this improvement approach?**

- Urology attendings / urology residents / Advanced Practice Providers
- Nurses and nurse managers depending on the site

1. **We will track the implementation of this improvement approach while it’s delivered**

**For educational meetings:**We recommend that every site at least:

- Discusses Modules 1 & 2 at least once

**For Champions:**

We recommend that the champion at least:

- Was identified and trained
- Raised awareness about the guidelines for appropriate frequency of surveillance cystoscopy procedures
- Articulated that appropriate frequency of surveillance cystoscopy procedures is high priority area for all team members and that it is consistent with existing regulations/rules/policies
- Reviewed improvement approaches (Module #2) content with local team members
- Provided access to the guidelines for appropriate frequency of surveillance cystoscopy procedures for the local team members
- Trained urology attendings / urology residents / Advanced Practice Providers on using templates / surveillance grids / educational materials
- Discussed the appropriate frequency of surveillance cystoscopy procedures based on risk with urology attendings / urology residents / Advanced Practice Providers
- Encouraged urology attendings / urology residents / Advanced Practice Providers to change existing frequency of surveillance cystoscopy procedures to be consistent with guideline recommendations

1. **Please list any tasks not listed above (i.e. modifications) you have taken while implementing this improvement approach (What? Why? Where? When? How? Who?):**

Observed benefits or drawbacks of tasks not listed above (i.e. modifications) while implementing this improvement approach:

**REMIND CLINICIANS:**

**What is happening?** This improvement approach is designed to help clinicians recall information and to prompt clinicians to appropriately assess bladder cancer risk and provide the corresponding appropriate frequency of surveillance cystoscopy procedures. It includes cheat sheets, posters, and changes to the electronic health record, such as easy access to guideline recommendations and standardizing documentation.

**Why is it happening?** This improvement approach may make it easier for clinicians to make the correct risk assessment and assign the appropriate frequency of surveillance cystoscopy procedures.

**Where is this happening?** Cheat-sheets, posters, and templates will be disseminated and implemented at your site.

**When will this happen? And for how long?** We anticipate that this improvement approach will start at your site on:      . The goal is to continue reminders for at least 6 months.

**How is this happening?**
In this section, we will specify the tasks that our team will do and the ones that you are expected to carry out at your local site. We will specify the items that must be completed to meet the minimum criteria for implementation of this improvement approach at your site.

1. **What would we like to accomplish?**

- Remind clinicians of guideline recommendations and increase awareness
- Provide easy access to guidelines
- Standardize documentation of the risk assessment, the clinical reasoning behind such assessment (the pathological features/ recurrence), the appropriate frequency of surveillance cystoscopy procedures, and which guideline is used (e.g., AUA guideline)
- Remind clinicians to inquire about social support needs and standardize documentation of those needs

1. **How are we going to help your site meet these goals?**

- Provide local team members access to the guidelines via cheat sheets, posters, and template in the electronic record
- Help with educating the local team members on the cheat sheets, posters, and template
- Communicate with champion/site PI to receive feedback about the cheat sheets, posters, and template
- Use the feedback from champion/site PI to improve the cheat sheets, posters, and template
- Discuss with site PI/champion the best way to inquire about and document social support needs

1. **Who’s in charge of implementing:
   the cheat sheets at your site?**

Site PI  Champion  Delegate [please specify who and for what task]:

**the posters at your site?**

Site PI  Champion  Delegate [please specify who and for what task]:

**the template at your site?**

Site PI  Champion  Delegate [please specify who and for what task]:

1. **Tasks to complete at your site to meet the goals of this improvement approach:
   Please identify which activities occurred at your site [check all that apply].**

Distribute cheat sheets and posters to local team members.

Place cheat sheets in easily accessible location.

Display poster in high visibility area.

Contact Clinical Application Coordinators (CACs) to integrate templates into the electronic health record and send them the template

Have a discussion with the local team members on how to identify and address social support needs (i.e., who is the gatekeeper and where do referrals go?) – this can be done in educational meeting (central research team will provide materials to help with this)

Educate local team members about templates and their use – this can be done in educational meeting (central research team will provide materials to help with this)

Make local adaptations to the template with CACs (e.g., how bladder cancer history is documented, whether social support needs are part of the template, and where social support referrals will go)

Troubleshoot the template with CACs as needed

Link template to cystoscopy note so this opens automatically

Trial template with test patient in local electronic health record

Share template that is being used at your site with the central research team when it is ready for use

If the template is modified, submit updated versions to the central research team

Provide usability feedback on cheat sheets and poster to research team (residents might be a good source for this feedback)

Provide usability feedback on template to research team (i.e., granular feedback such as missing fields, too many clicks, etc. Residents might be a good source for this feedback)

1. **Who would benefit from this improvement approach?**

- Urology attendings / urology residents / Advanced Practice Providers
- Nurses
- System (Electronic Health Record)

1. **We will track the implementation of this improvement approach while it’s delivered**We recommend that every site at least:

- Placed cheat sheets in easily accessible location
- Displayed poster in high visibility area
- Has a functional template that is shared with central research team and includes:
  - Actual text about guideline recommendations and link to guideline
  - Each patient’s risk-classification into low, intermediate, or high risk
  - Documentation of the appropriate frequency of surveillance cystoscopy procedures
- Educated local team members about templates and their use

1. **Please list any tasks not listed above (i.e. modifications) you have taken while implementing this improvement approach (What? Why? Where? When? How? Who?):**

Observed benefits or drawbacks of tasks not listed above (i.e. modifications) while implementing this improvement approach:

**PREPARE PATIENTS TO BE ACTIVE PARTICIPANTS:**

**What is happening?**  Our team will work with you (as local leaders and champions) to improve patients’ understanding of their surveillance plan by handing them surveillance grids. These surveillance grids outline the expected frequency of surveillance cystoscopy procedures and answers to frequently asked questions.

**Why is it happening?** A patient-facing improvement approach may help increase the rate of adherence, serve as a prompt to discuss surveillance schedules and risks, and facilitate scheduling and provision of social support as needed.

**Who receives the surveillance grids?** Patients with negative surveillance cystoscopy procedures. Patients with positive surveillance cystoscopy procedures will not receive the surveillance grids (because resection and pathology are needed for risk assessment).

**Where is this happening?** At your site, you will hand out the surveillance grids in (e.g., procedure room, post-procedure recovery area):       .

**When will this happen? And for how long?** We anticipate that this improvement approach will start at your site on:      . The surveillance grids will be handed out after the surveillance cystoscopy procedure and before the patient is sent home. The goal is to continue use of the surveillance grids for at least 6 months.

**How is this happening?**
In this section, we will specify the tasks that our team will do and the ones that you are expected to carry out at your local site. We will specify the items that must be completed to meet the minimum criteria for implementation of this improvement approach.

1. **What would we like to accomplish?**

- Ensure that patients undergo and adhere to the appropriate frequency of surveillance cystoscopy procedures
- Integrate explanation of bladder cancer risk and appropriate frequency of surveillance cystoscopy procedures into workflow
- Address patients’ concerns related to social support
- Address patients’ concerns related to their frequency of surveillance cystoscopy procedures

1. **How are we going to help your site meet these goals?**

- Our team developed surveillance grid prototypes that include:
  - bladder cancer risk
  - recommended surveillance schedule / grid
  - clear instructions to schedule before they leave the clinic if appropriate for your site
  - prompt to discuss social support and referral to social work as needed

1. **Who’s in charge of implementing the surveillance grids at your site?**

- To initiate the improvement approach:  Site PI  Champion  Delegate [please specify who and for what task]:
- To deliver the surveillance grids while interacting with patient:

Urology attendings / urology residents / Advanced Practice Providers  Nurses

1. **Tasks to complete at your site to meet the goals of this** **improvement approach:**

For this improvement approach there will be tasks for the Site PI/Champion or their delegate, and another set of tasks for local team members during their interaction with patients. **Please identify which activities occurred at your site [check all that apply]:**

1. For Site PI/Champion or their delegate:

Find space that is easy to access where the surveillance grids are stored

Have a pen / marker available at that space

Distribute the surveillance grid in clinic rooms/procedure rooms

Educate urology attendings / urology residents / Advanced Practice Providers and nurses on how to use the surveillance grids

Educate urology attendings / urology residents / Advanced Practice Providers and nurses on what to do if patients need social support

Encourage urology attendings / urology residents / Advanced Practice Providers to collect feedback about the grids from their patients

Provide the patient feedback to the central research team

Submit any modifications to the original grids to the central research team

1. For urology attendings / urology residents / Advanced Practice Providers / nurses:

For all patients with negative surveillance cystoscopy procedure:

Make the correct risk assessment and give the patient the corresponding surveillance grid

Discuss the surveillance grid with the patient and address any questions they may have

1. **Who would benefit from this improvement approach?**

- Urology attendings / urology residents / Advanced Practice Providers / nurses
  (benefiting from tasks in section 4.a)
- Patients (benefiting from tasks in section 4.b)

1. **We will track the implementation of this improvement approach while it’s delivered**We recommend that every site at least:

- Distributed surveillance grids in clinic rooms/procedure rooms for local team members’ use that contain:
- grid with schedule
- some space to address social support needs
- prompt/space for questions

1. **Please list any tasks not listed above (i.e. modifications) you have taken while implementing this improvement approach (What? Why? Where? When? Who? How?):**

Observed benefits or drawbacks of tasks not listed above (i.e. modifications) while implementing this improvement approach:

**ONGOING IMPROVEMENT & SUSTAINABILITY:**

- The goal is for local teams to continue improving risk-aligned bladder cancer surveillance as part of their usual processes
- Leadership is provided by the local leader / champion
- Towards the end of the 6-months of external facilitation, the central research team will discuss ways the local team can sustain successful practices
- The central research team will remain available for questions that may arise but will no longer regularly contact or meet with the team
- The central research team will reach out to collect final feedback on the improvement approaches after the external facilitation has ended

Please check any improvement approaches that you are planning to continue to use moving forward:

Review data to assess facility-level rate of appropriate frequency of cystoscopy procedures

Educational meetings focused on risk-aligned surveillance

Cheat sheets for risk-aligned surveillance

Posters summarizing bladder cancer risk and risk-aligned surveillance

Bladder cancer surveillance template in the EHR

Surveillance grids for bladder cancer patients

**2. Examples of the “Surveillance Grids” for low-, intermediate-, and high-risk bladder cancer.**

The following pages show the surveillance grids. They were printed two-sided, with the backside providing space for patients to note questions for their clinician.

**LOW Risk** Bladder Cancer – Surveillance

Name:

SS#:

Age:

- Your bladder cancer has been classified as **low** risk.
- Below is your follow-up surveillance plan.
- Please bring this to your next appointment.

First diagnosis

Most recent diagnosis (Stage, Grade, Date):

- After cystoscopy, you may have temporary burning or bleeding on urination.

(This should resolve within 3-4 days. Keep yourself well hydrated to help with this)

- For urgent concerns, please call the contact [space for phone number] or go to the ER.

*Today’s Date:*

**Questions for my Doctor:** Discussed

__________________________________________________________________________________________________________

__________________________________________________________________________________________________________

__________________________________________________________________________________________________________

__________________________________________________________________________________________________________

__________________________________________________________________________________________________________

Challenges for me to make it to appointments:

Remembering

Transportation

Work

Other obligations: _____________________________________

**INTERMEDIATE Risk** Bladder Cancer – Surveillance

Name:

SS#:

Age:

- Your bladder cancer has been classified as **intermediate** risk.
- Below is your follow-up surveillance plan.
- Please bring this to your next appointment.

First diagnosis

Most recent diagnosis (Stage, Grade, Date):

- After cystoscopy, you may have temporary burning or bleeding on urination.

(This should resolve within 3-4 days. Keep yourself well hydrated to help with this)

- For urgent concerns, please call the contact [space for phone number] or go to the ER.

*Today’s Date:*

**Questions for my Doctor:** Discussed

__________________________________________________________________________________________________________

__________________________________________________________________________________________________________

__________________________________________________________________________________________________________

__________________________________________________________________________________________________________

__________________________________________________________________________________________________________

Challenges for me to make it to appointments:

Remembering

Transportation

Work

Other obligations: _____________________________________

**HIGH Risk** Bladder Cancer – Surveillance

Name:

SS#:

Age:

- Your bladder cancer has been classified as **high** risk.
- Below is your follow-up surveillance plan.
- Please bring this to your next appointment.

Most recent diagnosis (Stage, Grade, Date):

First diagnosis

- After cystoscopy, you may have temporary burning or bleeding on urination.

(This should resolve within 3-4 days. Keep yourself well hydrated to help with this)

- For urgent concerns, please call the contact [space for phone number] or go to the ER.

*Today’s Date:*

**Questions for my Doctor:** Discussed

__________________________________________________________________________________________________________

__________________________________________________________________________________________________________

__________________________________________________________________________________________________________

__________________________________________________________________________________________________________

__________________________________________________________________________________________________________

Challenges for me to make it to appointments:

Remembering

Transportation

Work

Other obligations: _____________________________________

**3. Appendix Table.** Implementation outcomes, definitions, and data sources.

| **Outcome** | **Definition** | **Data Source** |
| --- | --- | --- |
| *Quantitative* | | |
| Adoption | Clinical outcome:  (1) whether the clinician accurately assessed bladder cancer risk and  (2) whether the clinician recommended a guideline concordant surveillance interval | Chart abstraction |
| Fidelity | Whether the strategy was integrated at the site as intended | Tracking in the blueprint |
| Sustainability | Whether the strategy was maintained at the site | Site verbal report |
| Patient experience and acceptability | Patient experience was assessed by a single item question inquiring about “the amount of information [they] received today” and the scale ranged from “not enough” (score of 1) to “just right” (score of 4) to “too much” (score of 7).  Acceptability was assessed by a four-item scale assessing how acceptable the presentation of bladder cancer-related information was during their surveillance visit. The response scale ranged from “strongly disagree” (score of 1) to “strongly agree” (score of 7). Acceptability was categorized as a scale score of 6 or higher. | Survey |
| *Qualitative* | | |
| Acceptability | perception among local stakeholders that the strategy was palatable or likable | Interview |
| Appropriateness | perceived fit of the strategy | Interview |
| Feasibility | the extent to which the strategy could be successfully used | Interview |
| Urologist satisfaction | references the general service experience including such features as wait times, scheduling, office environment [7]    Site experience with the improvement approaches and their associated features (e.g., wait times, scheduling, office environment) in the project | Interview |
| Urologist suggestions for improvement | Suggestions how improvement approach could be made better | Interview |
| Sustainability | Reasons as to whether the strategy was maintained at the site | Interview |
| Time spent | Comments on how much time was spent or whether amount of time was reasonable | Interview |

**4. Interview guide for clinical staff interviews in Aim 3 ImpRaBS**

**Introduction Overview:**

1. Introduce myself
2. Thank participant for involvement
3. Explain process of interview
   - I will ask mainly open-ended questions to learn about your thoughts about the improvement approaches for risk-aligned bladder cancer surveillance at your VA Medical Center
   - Our conversation here is to remain confidential.
   - Read script

**Introduction Script**

Thank you very much for participating in today’s interview. Our goal is to understand how well the improvement approaches for risk-aligned bladder cancer surveillance that we tested at your VA Medical Center worked for you. Our focus is on what we call risk-aligned surveillance. What we mean by that is following patients with low-risk superficial bladder cancer less frequently with cystoscopy (sis-TOS-kuh-pee) and those with high-risk cancer more frequently. We believe that you can help us with important insights into how we can make the improvement approaches better. The interview will last approximately 30 minutes.

Your participation is entirely voluntary and confidential. Should you decide to decline the invitation, your decision will remain confidential. Participation today would include taking part in today’s interview. You may choose to not answer any or all questions and you may terminate the interview at any time.

The interview will be audio recorded for later analysis. Once the data has been collected, it will be stored on a secure firewall protected server. Your name or other identifiable information will be redacted prior to analyses.

If you do not wish to answer any specific question during this interview, please just state that you do not wish to do so.

If you have questions about this study, you should contact Dr. Lisa Zubkoff, whose contact information is on the study information sheet that was provided to you previously or I can give it to you now. If you have questions, you should get them answered before you proceed. Do you wish to participate in today’s interview?

**Any questions on what will happen during this interview?**

**TURN ON RECORDING DEVICE**

- Verify and record consent of the staff member to be recorded

*Today’s date is [Month.Day.Year]. This is [interviewer’s name] and I am speaking with participant [ID #]. For the record,* *do you consent to participate in today’s interview and to be recorded?*

*Thank you.*

**Opening:**

*Again, we are interested in ways to help your site implement risk-aligned surveillance, basically getting patients the surveillance they need according to the guidelines. For this project, we have developed four improvement approaches and your site has implemented them. The four are:*

1. *educational sessions covering bladder cancer risk assessment and guideline recommendations along with an overview of the improvement approaches*
2. *reminders in the form of a CPRS templates, cheat sheets, and posters [can show examples of those TEAMS]*
3. *a surveillance grid to be handed to patients [can show example of this via TEAMS]*
4. ***[for providers]****: support from your local site leader or champion****[for champions]****: support from the central research team*

*We are interested in your thoughts on the current project and these four improvement approaches.*

***A*** *What have you heard about this project?*

***[If they have heard about it]****:***🡪 GO TO SECTION B**

***[If they have not heard about it]****:
1) Have you seen a bladder cancer template / poster / cheat sheet / or surveillance grid at your site?
2) Was the topic of risk-aligned bladder cancer surveillance discussed in any team meetings / ad-hoc clinic interactions / or grand rounds at your site?****🡪 If yes to either of the above 2 questions, go to 2a). If no to both, go to 2b).***

1. ***[If YES]:*** *It may not have been clearly communicated to you, but these are actually the improvement approaches that we are interested in and want to talk to you about.****🡪 GO TO SECTION B***
2. ***[If NO]:*** *OK, in general what are your thoughts on providing risk-aligned surveillance for patients with superficial bladder cancer?*
   *What kind of support would you recommend for a VA site that is struggling to offer risk-aligned bladder cancer surveillance?
   How much does your local VA leadership focus on providing risk-aligned bladder cancer surveillance?
   Tell me about any discussions or communications you may have had about risk-aligned bladder cancer surveillance with colleagues?****[END OF INTERVIEW].***

**B Now we would like to talk about some of the approaches. Of the 4 improvement approaches, education, reminder tools such templates/posters/cheat sheets, surveillance grid, and support from research team or champion, which had the *most impact? Why?***

**[education / reminders / surveillance grid / support from research team or champion]**

1. *How does [most impactful approach] help you and your team members with providing risk-aligned bladder cancer surveillance? [usability, helpful?]*
2. *Why was the [most impactful approach] suitable for your site? [appropriateness]*
3. *What aspects of [most impactful approach] do you like? [acceptability]*
4. *How easy was it to start using [your most impactful approach] at your site? [feasibility]*
5. *Do you have any suggestions on how [most impactful approach] could be improved?*
6. ***[for providers]****: How reasonable was the amount of time you spent [receiving education OR support] OR [using the templates/posters/cheat sheets, OR surveillance grids]? [time spent]****[for champions]****: How reasonable was the amount of time you spent on [providing education OR support] OR [adapting and implementing the templates/posters/cheat sheets, OR surveillance grids] [time spent]*

**C So you told us [improvement approach] had the most impact. If you were to rank the improvement approaches from most impactful to least impactful, which one would you put on the bottom of the list?** *[Be persistent and frame it as a ranking exercise. If participant is adamant or refuses to categorize any as “least impactful” move onto next set of questions.]* **Why do you think this ranks lowest on the list?**

**[education / reminders / surveillance grid / support from research team or champion]**

1. *How suitable is/are the [least impactful approach] for your site? [appropriateness]*
2. *How easy was it to start using [your least impactful approach] at your site? [feasibility]*
3. *Even though it was the least impactful, how could [least impactful approach] help other team members with providing risk-aligned bladder cancer surveillance? [usability, helpful?]*
4. *Even though it was the least impactful, what aspects of [least impactful approach] might other team members like? [acceptability]*
5. *Do you have any suggestions on how [least impactful approach] could be improved?*
6. ***[for providers]****: How reasonable was the amount of time you spent [receiving education OR support, OR using the templates/posters/cheat sheets, OR surveillance grids]? [time spent]****[for champions]****: How reasonable was the amount of time you spent on [providing education OR support, OR adapting and implementing the templates/posters/cheat sheets OR surveillance grids] [time spent]*

**D Now that you have told us about the most and least impactful approach, which of the four approaches would you recommend to another VA site? Why?**

**[education / reminders / surveillance grid / support from research team or champion]**

**E Now we are interested in talking about data on how much risk-aligned bladder cancer surveillance your site provides.**

1. *What have you heard about data on documentation of risk assessment at your VA? [feasibility/ usability]*
2. *What have you heard about data on risk-aligned frequency of surveillance at your VA? [feasibility/ usability]*
3. ***[If they have heard about either #1 or #2]:*** *- From whom and how did you hear about it?
   - Did this data help you and your team members with providing risk-aligned bladder cancer surveillance? [usability, helpful?]
   - As you may know, the data provided was averaged across urology providers at your VA? How suitable was this for improving risk-aligned surveillance at your site? [appropriateness]
    Prompt: Would data at the provider level more suitable?
   - Do you have any suggestions how we can improve the delivery of data on risk-aligned bladder cancer surveillance?****🡪 GO TO SECTION F***



1. ***[If they have not heard of both #1 and #2]:*** *- Would it be helpful to receive averaged data on risk-aligned bladder cancer surveillance across urology providers at your VA?
    Prompt: Would data at the provider level be more suitable?****🡪 GO TO SECTION F***

**F To wrap up, we would like you to think globally about the improvement approaches.**

1. *How did the improvement approaches help you implement risk-aligned surveillance at your VA? [Feasibility of risk-aligned surveillance]
   Prompts:
    -* ***[If they have not talked about education yet]:*** *How about the impact of educational meeting?
   - [****If they have not talked about reminders yet]:*** *How about the impact of reminders, such as the template, cheat-sheet, posters?*
   *-* ***[If they have not talked about the surveillance grid yet]:***  *How about the impact of patient involvement by using surveillance grids?
   -* ***[If they have not talked about support from the research team or champion yet]:*** *How about the impact of support from the research team or champion?
    -* ***[for champions only]****: How about the impact of the blueprint? How about the impact of regular facilitation meetings?*
2. *Now that you experienced the different improvement approaches, to what extent do you think that risk-aligned surveillance is the right care for your bladder cancer patients? [acceptability of risk-aligned surveillance]*

**Closing questions:**

*We have been talking about your thoughts, experiences, and suggestions regarding the improvement approaches for risk-aligned bladder cancer surveillance.*

*1. Have we missed anything that is important to you as you think about your experiences with the improvement approaches and risk-aligned bladder cancer surveillance?*

*2. Would you recommend the improvement approaches used in this project to your colleagues at other VA Medical Centers to improve risk-aligned bladder cancer surveillance? Why?*

***Thank you for participating.***

**5. Codebook**

| **Domain** | **A priori code** | **Definition** | **Coding Instructions** |
| --- | --- | --- | --- |
| **Implementation Outcomes** | | |  |
|  | Acceptability | Perception that a specific improvement approach is satisfactory. Include positive and negative responses. Use when "Helpful" is mentioned when tied to an approach (e.g., educational sessions). Do NOT double code with Appropriateness or Feasibility. | Comments about a specific improvement approach and aspects they like/don't like. Include positive and negative responses. Words such as: approval, appealing, liked, welcomed |
|  | Appropriateness | Perceived fit or relevance of improvement approach for a given setting, provider, or consumer. Include positive and negative responses. Use when "Helpful" is mentioned and when not linked to other terms (e.g., liked, welcomed). Do NOT double code with Acceptability or Feasibility | Does the improvement approach fit or is it "suitable" in a specific setting or with a provider/patient? Include positive and negative responses. Words such as: helpful, fitting, suitable, applicable, good match |
|  | Feasibility | Extent to which a new treatment or innovation can be successfully used or carried out within given setting. Include challenges with using approaches. Do NOT double code with Acceptability or Appropriateness | How realistic or easy is using/implementing an approach or surveillance? Include challenges with using approaches. |
|  | Challenges | Challenges with ImpRaBS or improvement approaches without suggestions for improvement. |  |
|  | Satisfaction | Participants' overall perception and experience of the ImpRaBS program and clinical services (e.g., wait times, funding, scheduling, etc.) | Perception and experience with overall ImpRaBS program and clinical services (e.g., protected time, funding, global impact on service reputation, wait times, scheduling, etc.) |
|  | Suggestions | Suggestions to improve approaches, actionable changes or discussions of challenges that reference the potential to increase effectiveness of approaches | None, follow definition shown to the left. |
|  | Time spent | How much time was spent on [improvement approach or implementing ImpRaBS]? Was that amount of time reasonable? | None, follow definition shown to the left. |
|  | Sustainability | Will [improvement approach] be used in the future? | None, follow definition shown to the left. |
| **Implementation Strategy** | |  |  |
|  | Educational sessions | Educational sessions covering risk assessment, guideline recommendations, and improvement approaches (#1) | None, follow definition shown to the left. |
|  | Reminders | Overarching term for all reminders. | Reminders in the form of CPRS templates, cheat sheets, and posters (#2) |
|  | Reminder: Cheat sheet | Reminder in form of cheat sheet (#2) | None, follow definition shown to the left. |
|  | Reminder: Poster | Reminder in form of poster (#2) | None, follow definition shown to the left. |
|  | Reminder: Template | Reminder in form of template, referred to as CPRS template (#2) | None, follow definition shown to the left. |
|  | Surveillance grid | Improvement approach #3: Surveillance grid handed to patients (#3) | None, follow definition shown to the left. |
|  | Central research team support | Support from central research team from champions (#4) | For champions only: Support from central research team from champions (#4) |
|  | Champion support | Support from local site leader or champion from providers (#4) | For providers only: Support from local site leader or champion from providers (#4) |
|  | Audit and feedback | Did they see data on providers' performance and risk-aligned surveillance? Was it helpful? Only code if they discuss data shared with them during the ImpRaBS project. IF they received this outside of project, then code as Suggestion | None, follow definition shown to the left. |
|  | Implementation Blueprint | Document used to track implementation activities during pilot implementation (champion) | For champions only: Document used to track implementation activities during pilot implementation |
|  | Tailored strategies | Were any approaches tailored to meet the local context NOT due to changes in evidence or guidelines? Not discussions of future changes to approaches | None, follow definition shown to the left. |

**6. Survey collected from Veterans in Aim 3** (Adapted from Weymiller AJ, Montori VM, Jones LA, et al: Helping patients with type 2 diabetes mellitus make treatment decisions: statin choice randomized trial. Arch. Intern. Med. 2007; 167: 1076–1082.)

Thank you for agreeing to take the time to inform **our efforts to improve risk-aligned bladder cancer surveillance**. Your responses to this brief questionnaire will be kept confidential.

Please rate your view of your bladder cancer follow-up visit today.

|  | |  | Not enough |  |  | | Just right | |  | | |  | Too much |
| --- | --- | --- | --- | --- | --- | --- | --- | --- | --- | --- | --- | --- | --- |
| 1. | The amount of information I received today was: | | □ | □ | □ | | □ | | □ | | | □ | □ |
|  |  | | Strongly disagree |  |  | | Neutral | |  | | |  | Strongly agree |
| 2. | The information I received today was clear and easy to understand. | | □ | □ | □ | | □ | | □ | | | □ | □ |
| 3. | The information I received today was helpful. | | □ | □ | □ | | □ | | □ | | | □ | □ |
| 4. | I would recommend the way bladder cancer care was discussed with me to others. | | □ | □ | □ | | □ | | □ | | | □ | □ |
| 5. | I would like to receive information about other care or procedures in the same way as today. | | □ | □ | □ | | □ | | □ | | | □ | □ |
|  |  | |  |  | |  | |  | |  |  | |  |

**If you have any additional comments about bladder cancer follow-up visit today, please note them here:**

**_**

**_**

**_**

**7. Appendix Figure 1.** Plots of the timelines for integrating the template, blueprint, and educational meetings across four sites. Time zero represents the implementation of the strategy. The width of the bar describes the time from when integration of the strategy was started to its availability for clinicians to use. For example, a bar that starts at approximately -180 days means that it took about 180 days from when discussions began to when clinicians had access. In some cases, strategies were used as soon as they were available.


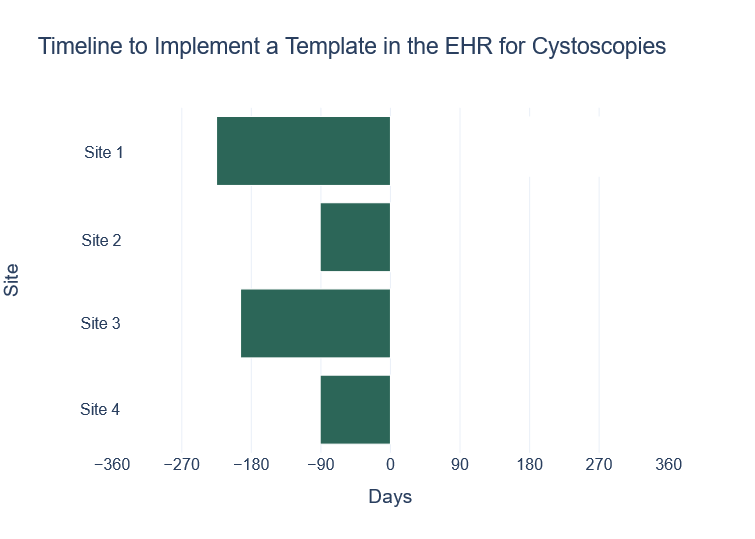


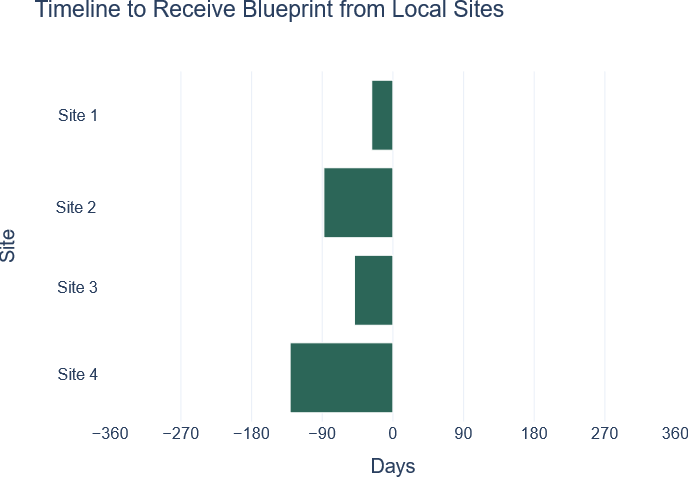


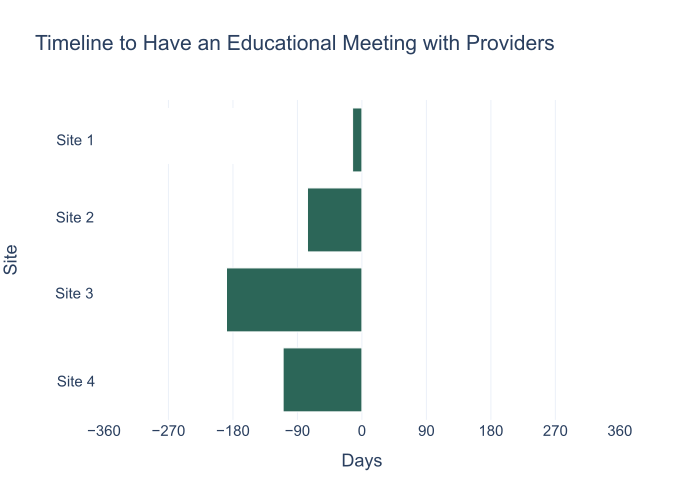

Supplement: Supplementary file 1 — Additional file 1. [file 43058_2025_721_MOESM1_ESM.docx]
